# Spatial distribution of Culex mosquito abundance and associated risk factors in Hanoi, Vietnam

**DOI:** 10.1371/journal.pntd.0009497

**Published:** 2021-06-21

**Authors:** Tuyen V. Ha, Wonkook Kim, Thang Nguyen-Tien, Johanna Lindahl, Hung Nguyen-Viet, Nguyen Quang Thi, Huy Van Nguyen, Fred Unger, Hu Suk Lee

**Affiliations:** 1 Faculty of Resources Management, Thai Nguyen University of Agriculture and Forestry (TUAF), Thai Nguyen, Vietnam; 2 Pusan National University, Busan, South Korea; 3 International Livestock Research Institute (ILRI), Hanoi, Vietnam; 4 Department of Medical Biochemistry and Microbiology, Uppsala University, Uppsala, Sweden; 5 Department of Clinical Sciences, Swedish University of Agricultural Sciences, Uppsala, Sweden; Centers for Disease Control and Prevention, Puerto Rico, UNITED STATES

## Abstract

Japanese encephalitis (JE) is the major cause of viral encephalitis (VE) in most Asian-Pacific countries. In Vietnam, there is no nationwide surveillance system for JE due to lack of medical facilities and diagnoses. *Culex tritaeniorhynchus*, *Culex vishnui*, and *Culex quinquefasciatus* have been identified as the major JE vectors in Vietnam. The main objective of this study was to forecast a risk map of *Culex* mosquitoes in Hanoi, which is one of the most densely populated cities in Vietnam. A total of 10,775 female adult *Culex* mosquitoes were collected from 513 trapping locations. We collected temperature and precipitation information during the study period and its preceding month. In addition, the other predictor variables (e.g., normalized difference vegetation index [NDVI], land use/land cover and human population density), were collected for our analysis. The final model selected for estimating the *Culex* mosquito abundance included centered rainfall, quadratic term rainfall, rice cover ratio, forest cover ratio, and human population density variables. The estimated spatial distribution of *Culex* mosquito abundance ranged from 0 to more than 150 mosquitoes per 900m^2^. Our model estimated that 87% of the Hanoi area had an abundance of mosquitoes from 0 to 50, whereas approximately 1.2% of the area showed more than 100 mosquitoes, which was mostly in the rural/peri-urban districts. Our findings provide better insight into understanding the spatial distribution of *Culex* mosquitoes and its associated environmental risk factors. Such information can assist local clinicians and public health policymakers to identify potential areas of risk for JE virus. Risk maps can be an efficient way of raising public awareness about the virus and further preventive measures need to be considered in order to prevent outbreaks and onwards transmission of JE virus.

## Author summary

Our study was to estimate the spatial distribution of *Culex* mosquitoes in Hanoi, which is one of the most densely populated cities in Vietnam. Our findings may provide better insight into understanding the spatial distribution of *Culex* mosquitoes and its associated environmental risk factors. Such information can assist local clinicians and public health policymakers to identify the potential areas of risk for JE virus.

## Introduction

Japanese encephalitis (JE) is the major cause of viral encephalitis (VE) in most Asian-Pacific countries. It is a vector-borne flavivirus, in the same viral family, *Flaviviridae*, as dengue, yellow fever and West Nile virus [1–3]. About 3.1 billion people live in endemic areas with an estimated number of 50,000–68,000 clinical cases and 10,000–15,000 deaths annually [4,5]. Most human infections are asymptomatic, or very mild febrile disease, but when clinical encephalitis occurs, the death rate could reach 30%, and another 30–50% of patients suffer from permanent neurologic or psychiatric sequelae [4,6].

In Vietnam, the JE virus was first reported in 1951 and became a serious public health issue across the country, particularly in the northern part [7]. Currently, there is no nationwide surveillance system for JE due to lack of medical facilities and diagnoses, but there is national surveillance data available for viral encephalitis (VE) [8]. Some studies found that JE cases accounted for 12–72% of total VE cases, therefore VE cases can be considered to be a proxy for JE cases [8–10]. Before the vaccination was scaled out, the Red River Delta region (including Hanoi) had the highest JE incidence rate with an estimated 22 cases /100,000 people [8]. *Culex tritaeniorhynchus*, *Culex vishnui*, and *Culex quinquefasciatus* have been identified as the major JE vectors in Vietnam, of which the two first were mosquitoes associated with rice production, pig farms and wastewater [11–13]. In Hanoi, a study between 2006 and 2008 found the most *Culex* mosquitoes in rural districts of the city, and also isolated three JE virus strains from mosquitoes in the southern and western parts of Hanoi [14]. Pigs are a major amplifying host for the JE virus, and since they are commonly raised in the backyard farms, the transmission of the virus to humans is facilitated [5,15]. A study conducted in Hanoi showed that approximately 60% of pig samples from slaughterhouses were seropositive [16].

Remote sensing, with recent advances in sensor capability and open-access data policies, can provide great opportunities for public health researchers and scientists to predict and map the abundance of mosquitoes or disease risks [17–19]. By linking data from the known mosquito abundance locations with their associated environmental and socio-economic information, researchers can estimate the mosquito species distribution in other less well known locations [20]. Several studies have been conducted to map the abundance of different *Culex* species and potential JE transmission risk areas worldwide [21–23]. Studies suggest that the Asia, including the Southeast Asia region have the highest risk of *Culex* mosquitoes whereas approximately 1.7 billion people are estimated to live in areas with a high risk of encephalitis [24–26].

Previous studies have been designed to estimate the mosquito distribution at the regional and global level, which may not be suitable for the local context. In Vietnam, some studies have sought to understand the ecology of JE vectors and *Culex* mosquitoes [12,27,28]. However, to our knowledge, no studies have been conducted to estimate the spatial distribution of *Culex* mosquito abundance in Vietnam using remote sensing data. Therefore, the main objective of this study was to estimate the spatial distribution of *Culex* mosquitoes in Hanoi, which is one of the most densely populated cities in Vietnam. This information can be helpful for local health authorities and clinicians to implement better JE control programs by identifying the potential hotspot areas for the virus.

## Materials and methods

### Ethics statement

This study was approved by the Institutional Review Board for Biomedical Research of Hanoi University of Public Health No. 406/2018 YTCC-HD3.

### Study area and mosquito data

**Fig 1:**
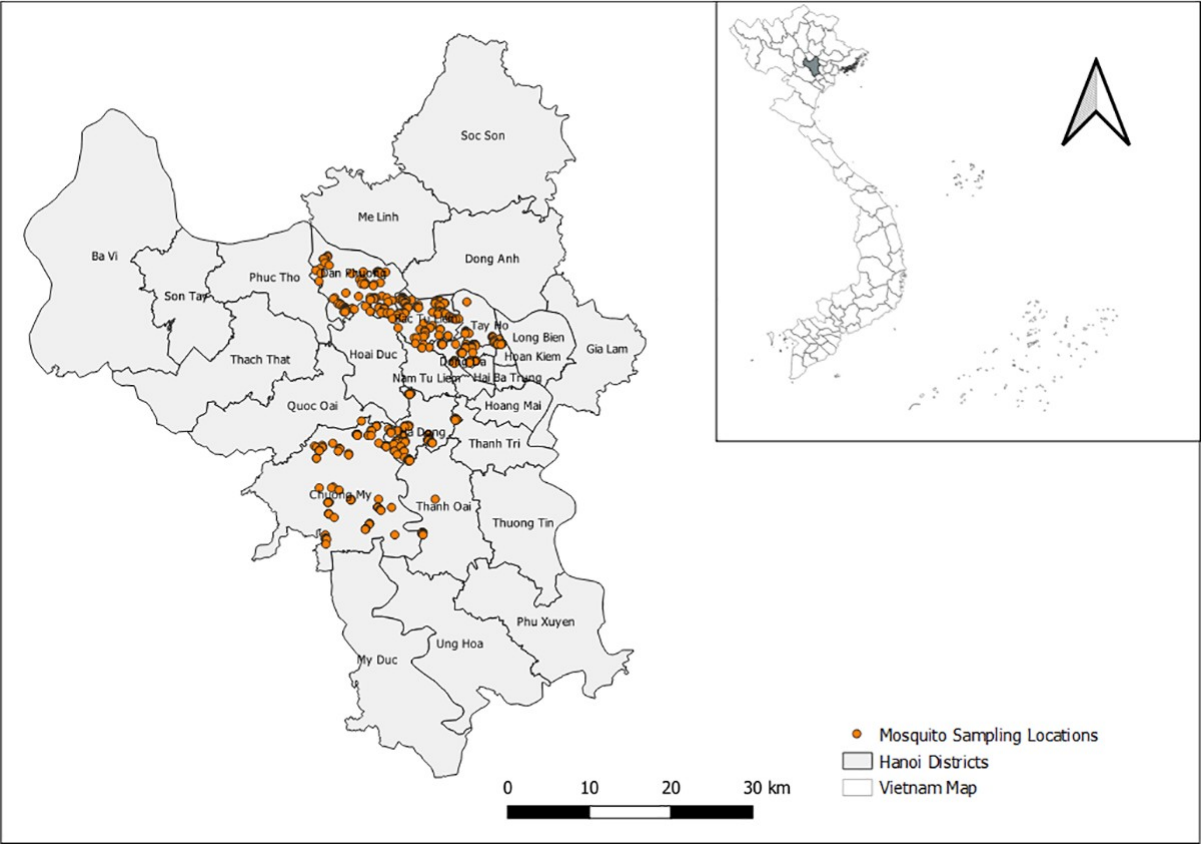
The study area with mosquito sampling locations in Hanoi (source of shapefile: https://www.diva-gis.org/gdata).

The study location was Hanoi, the capital of Vietnam, which has an area of 3.359km^2^, consists of 30 districts and is home to approximately 8 million people (Fig 1).

The city’s landscapes are mainly characterized by cropland and built-up infrastructure with most of the land below 20m above sea level [29]. Although the study area has experienced rapid expansion of urbanization, the majority of the land is occupied by agriculture farms (~40%), especially rice fields [30]. The rainfall and temperature vary significantly between months with monthly mean ranges of 16-330mm and 17–29°C, respectively, with a peak between June and August. The subtropical climate along with extensive rice production ecosystems can create favorable conditions for mosquito breeding [12,31].

Mosquito samples were collected in urban and peri-urban/rural areas of Hanoi from September to October 2018. A total of 513 locations were randomly selected for mosquito trapping in six districts, and the nearest consenting household with livestock and without livestock was included. At each household, CDC light and BG sentinel traps were used to collect mosquitoes both in houses and outside/gardens. Traps in the garden were installed within a radius of 20-30m from the house by investigators. Households with no insecticides or mosquito sprays were selected to avoid sampling biases.

The collected mosquito samples were transported to the Vietnam National Institute of Hygiene and Epidemiology in cool containers and identified/grouped into species using taxonomic keys [11]. The number of *Culex* mosquitoes was counted by experts at each sampling location. Since all sampling locations were randomly selected, some traps were installed very close to each other. In this case, the total number of counted mosquitoes was aggregated as one point if more than two traps were located within the same pixel (30m). The analyses considered only female mosquitoes identified as *Cx*. *tritaeniorhynchus*, *Cx*. *quinquefasciatus*, and *Cx*. *vishnui* since these are the primary vectors for JE virus transmission [32].

### Environmental and socio-economic predictor variables

The survival and distribution of mosquitoes species can be significantly influenced by climate and socio-economic conditions [33,34]. For meteorological data, we collected temperature and precipitation information during the study period and its preceding month. In addition, the other predictor variables (e.g., normalized difference vegetation index [NDVI], land use/land cover and human population density), were collected for our analysis. The NDVI represents vegetation health and its value ranges from -1 to +1, a higher NDVI value means denser and healthier vegetation and vice versa [35]. All the collected data was pre-processed with the same study extent, resampled to the same spatial resolution of Landsat 8 (30m pixel size), and then converted to UTM zone 48N.

Specifically, we used eight-day average MODIS land surface temperature data from NASA’s Land Processes Distributed Active Archive Center (https://lpdaac.usgs.gov/), and precipitation data from the Climate Hazards Center (https://chc.ucsb.edu/data/chirps). Data included mean temperature (September and October), previous-month temperature (August), mean total rainfall (September and October), and previous-month rainfall (August). The 30m land cover/land-use layer with five classes (rice, urban/built-up, water body, forest and other agricultural land) was obtained from a previous study [36]. In addition, the human population density layer for 2015 was downloaded from the NASA SEDAC at the Center for International Earth Science Information Network while the mean NDVI was calculated from Landsat 8 (red and near-infrared bands) data (https://earthexplorer.usgs.gov/) during the study period.

Previous research suggested that mosquitoes are active within hundreds of meters (e.g. 200-300m) from their nearest breeding sites [37,38]. Also, most mosquito-borne diseases were observed among people living near mosquito breeding habitats [39]. Thus, we computed the cover ratios for rice, forest, and water at 250m buffer radius for every pixel whereas meteorological, human population and NDVI layers were taken as an average. Therefore, a total of 9 potential risk factors were used for developing a model.

### Data analysis

A Poisson regression model has been commonly used for countable data, which assumes the equality between the sample mean and variance [40,41]. However, our mosquito count data showed evidence of overdispersion (variance is greater than the mean). An alternative approach for over-dispersed count data is negative binomial regression (NBR) because it has an extra parameter (alpha [α]) to account for the over-dispersion [42, 43].

Given Y is the response variable (*Y*∈{0,1,2,3,4…}), representing the number of mosquitoes at a certain location, having *x*_*1*_, *x*_*2*_, *x*_*3*_, *…*., *x*_*i*_ with predictor variables (e.g. environmental and socio-economic variables). The NBR distribution can be expressed as follow [44, 45]:

$$\mathrm{Pr}\left(Y=y_{i}|\mu_{i},\alpha \right)=\frac{\Gamma (y_{i}+\alpha^{-1})}{\Gamma (\alpha^{-1})\Gamma (y_{i}+1)}{\left(\frac{1}{1+\alpha \mu_{i}}\right)}^{\alpha^{-1}}{\left(\frac{\alpha \mu_{i}}{1+\alpha \mu_{i}}\right)}^{y_{i}}$$

Model mean and variance are $E(y_{i})=\mu_{i}=e^{\beta_{0}+\beta_{1}x_{1}+\beta_{2}x_{i}+\cdots +\beta_{i}x_{i}}$ and $\mathrm{var}(y_{i})=\mu +{\alpha \mu }^{2}$, respectively, and α (α ≥ 0) is the dispersion parameter. To minimize the overfitting problem, our data was randomly split into two parts, training (80%) and testing (20%).

A shapefile is a digital vector storage format for storing geometric location and associated attribute information. We used the point vector shapefile (https://www.diva-gis.org/gdata) with mosquito count data in order to extract environmental and socio-economic values from associated raster layers using raster package in R [46]. We used “glmmTMB” package in R to evaluate the association between mosquito abundance and environmental predictor variables. The glmmTMB package is used to fit generalized linear mixed models with various extensions for random effects and non-normal data such as negative binomial regression as described by Magnusson et al [47]. For variable screening, all predictor variables were examined for their collinearity using Pearson’s correlation test and any variables with a Pearson’s coefficient <0.7 were considered in the models. If two or more variables were strongly correlated, we considered their biological relevance with mosquitoes. In addition, the effects of linearity between potential predictor variables and mosquito abundance were explored using loess smoothed curves. If any nonlinearity was observed, a quadratic function of the predictor variable was explored and retained if p-value <0.05.

Because of nonlinearity between mosquito abundance and total mean rainfall, we created two new variables (1. centered variable, 2. quadratic term variable) from the current month rainfall (September-October). The centered variable was computed by subtracting the rainfall mean across mosquito sampling locations while the quadratic term variable was the square of the centered variable. In addition, a random effect for sampling locations (rural/peri-urban and urban) was included to take into account the variability among trapping locations. Variables with p-value < 0.05 were considered in the final model. The incidence rate ratio (IRR) was calculated for the final NBR model.

The performance of the final fitted model was assessed using root mean square error (RMSE) and mean absolute error (MAE). The RMSE is the square root of the average of squared differences between predicted values and observed values while the MAE is the average of absolute differences between predicted values and observed values [48,49]. Finally, three additional raster layers (centered, quadratic term and random effect layers) were created and stacked with other relevant layers, and then the abundance of mosquitoes was estimated with stacked layers from the final model. All data analysis was conducted in R version 4.0.1 while risk maps were created through QGIS version 3.16.

## Results

### Mosquito descriptive statistics

**Fig 2:**
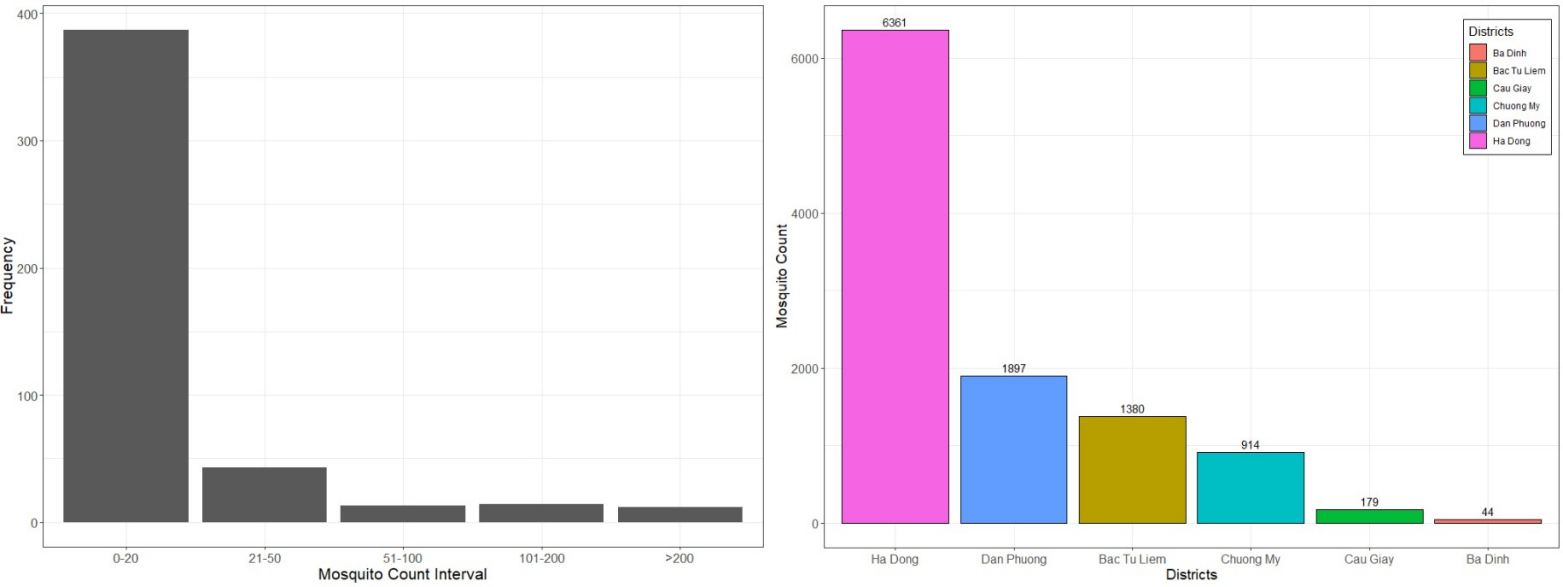
Distribution and mosquito sampling count from six districts in Hanoi.

A total of 10,775 female adult *Culex* mosquitoes were collected from 513 trapping locations. The most dominant *Culex* mosquitoes were *Cx*. *tritaeniorhynchus* (80%), followed by *Cx*. *quinquefasciatus* (10.39%) and *Cx*. *vishnui* (9.61%). Also, our mosquito data showed that nearly 97% of the samples had less than 150 cumulative counts per location during the study period whereas approximately 2% of trapping locations had a cumulative count of more than 200 mosquitoes (Fig 2, left).

A total of 6,361 mosquitoes were obtained in Ha Dong suburb district, accounting for 59% of the total while Ba Dinh (urbanized district) generated 44 collected mosquitoes (0.4%) (Fig 2, right). Overall, the rural/ peri-urban districts had a much higher number of mosquitoes than densely urban areas.

### Final NBR models and estimated spatial distribution of *Culex* abundance

Overall, the NDVI value and temperature (lag1) had a strong correlation with bi-monthly temperature whereas other remaining variables were less correlated (Table 1). The final model selected for estimating the *Culex* mosquito abundance included centered rainfall, quadratic term rainfall, rice cover ratio, forest cover ratio, and human population density variables. The NBR model showed that there was an increase in the mosquito abundance as total monthly rainfall rose to its mean value (220mm), but it dropped gradually as the precipitation increased beyond its mean value (Table 2). In addition, a percentage increase in rice cover ratio corresponded to an increase in the mosquito abundance by 3.78 times. In contrast, a 1% increase in forest cover ratio corresponded to a reduction in the mosquito abundance by 97.5%. Similarly, a unit increase in human population density (per km^2^) had resulted in 1.1% reduction of the mosquito population. Our final model showed that the RMSE and MAE were 24.15 and 16.83, respectively.

**Table 1:** Pearson’s correlation coefficient (r) among predictor variables.

Variables	Forest cover ratio (%)	Rice cover ratio (%)	Water cover ratio (%)	Temperature (lag1, ^o^C)	Bi-monthly temperature (^o^C)	Rainfall (lag1, mm)	Bi-monthly rainfall (mm)	NDVI	Human population density (per km^2^)
Forest cover ratio	1								
Rice cover ratio	0.24	1							
Water cover ratio	-0.13	0.10	1						
Temperature	-0.23	-0.51	-0.14	1					
Bi-monthly temperature	-0.29	-0.38	-0.16	0.84	1				
Rainfall	0.07	0.24	-0.03	-0.25	-0.14	1			
Bi-monthly rainfall	-0.18	0.01	-0.23	0.30	0.57	0.14	1		
NDVI	0.27	0.47	-0.03	-0.7	-0.48	0.37	-0.05	1	
Human population density	-0.21	-0.46	-0.06	0.62	0.35	-0.52	-0.06	-0.56	1

Lag1 = preceding month; bi-monthly = during study period

**Table 2:** Final NBR model with accuracy and associated risk factors of Culex mosquito abundance in Hanoi.

Variables	Adjusted IRRs	95% CI	P-value	RMSE	MAE
Bi-monthly total mean rainfall (mm, centering variable)	1.0492	1.021–1.077	<0.0004		
Bi-monthly total mean rainfall (mm, quadratic term)	1.001	1.000–1.002	0.0475		
Rice cover ratio (%)	3.778	1.549–9.204	0.0034		
Forest cover ratio (%)	0.0249	0.003–0.105	<0.0001		
Human population density (person per km^2^)	0.989	0.986–0.991	<0.0001		
**Accuracy**				24.15	16.83

CI = confidence interval; IRR = incidence rate ratio; NBR = negative binomial regression; Bi-monthly = study period (Sep-Oct)

**Fig 3:**
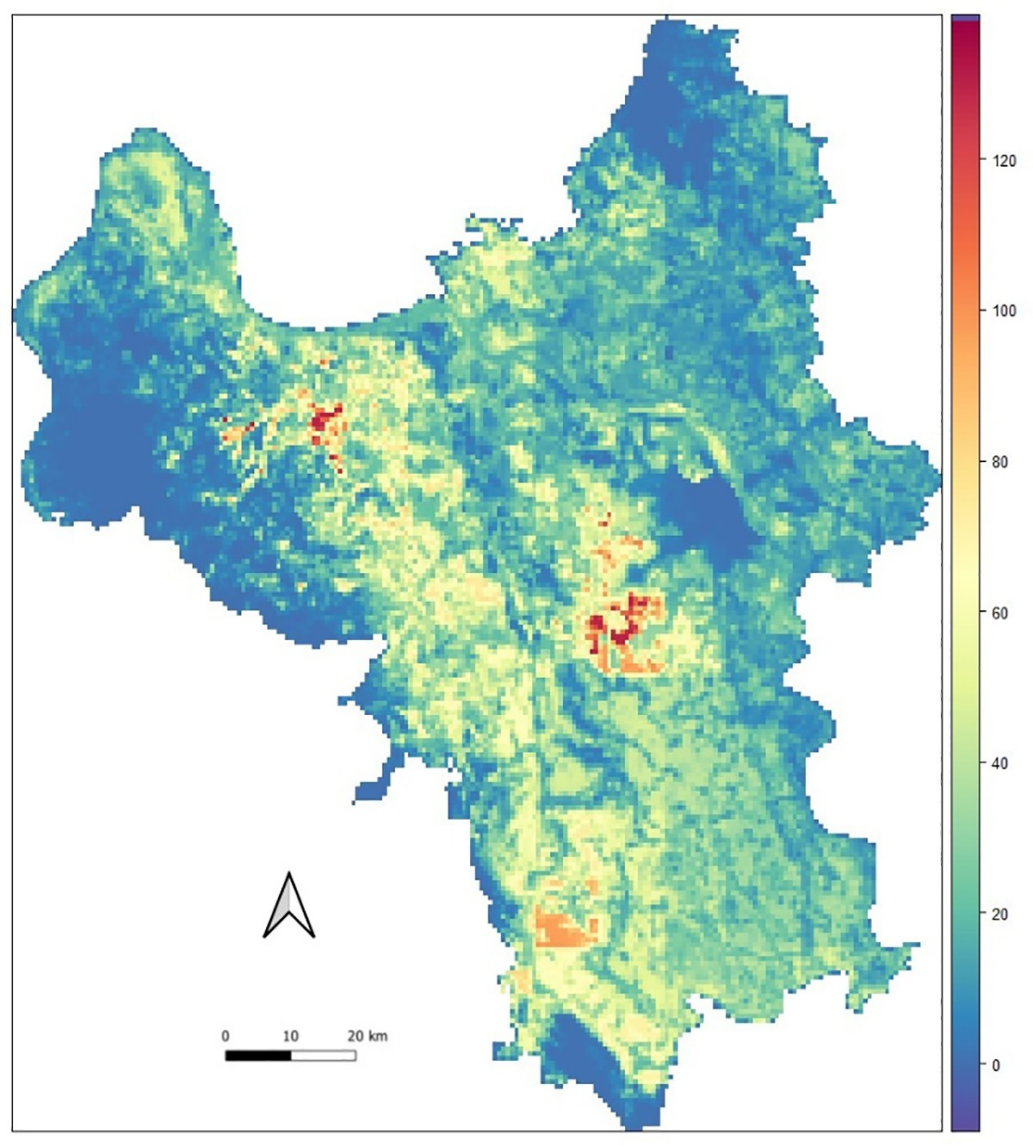
Estimated map of the Culex mosquito abundance in Hanoi (source of shapefile: https://www.diva-gis.org/gdata).

The estimated spatial distribution of *Culex* mosquito abundance ranged from 0 to 150, and the colors ranged from purple-blue for areas with low to orange-red berry for areas with high abundance of the mosquitoes (Fig 3).

The abundance of *Culex* species was mainly observed in rural and agriculture areas, whereas the central districts of Hanoi and forest areas showed the lowest abundance. Noticeably, some areas in the northwest region had the highest mosquito abundance. Our model estimated that 87% of the Hanoi area had an abundance of mosquitoes from 0 to 50, whereas approximately 1.2% of the area showed more than 100 mosquitoes, which was mostly in the rural/peri-urban districts.

## Discussion

This study was the first evaluation of the spatial abundance of *Culex* mosquitoes using remote sensing data in Hanoi, Vietnam. We found that the rice paddy cover was positively associated with the spatial distribution of mosquito abundance, which was similar to findings from previous studies, suggesting that the rice crop ecosystem is the most important breeding habitat for the *Culex* mosquitoes, and expanding rice cultivation is positively associated with JE virus. [12,13,50]. These results are similar to those of an earlier study that observed *Culex* mosquitoes in the rice fields and stagnant water habitats in northern Vietnam [12]. In addition, rainfall was identified as the main driver of *Culex* mosquito abundance in areas with rainfall level below 220mm, then gradually decreased at rainfall level higher than 220mm annually. A Chinese study found that a precipitation between 80mm and 120mm was suitable for mosquito growing and breeding. Other studies have suggested that locations with higher precipitation were more suitable for mosquitoes with JE virus, but excessive rainfall such as typhoons could wash away mosquitos’ habitats and larvae [51,52].

However, this study found that high forest cover had a negative association with mosquito abundance. One study, in Thailand, suggested that mosquitoes were least likely to survive in dense forests because of unfavorable environmental conditions (e.g. low temperature) [53]. Similarly, other studies found that a forest with bushy vegetation was likely to block the sunshine which reduced the temperature of small water bodies, thereby preventing larval growth [54,55]. Our study also exhibited that mosquito abundance decreased as human population density increased which was consistent with previous studies [56,57]. These studies indicated that the reduction of mosquitoes in more densely populated areas was due to regular mosquito control programs, improved urban planning, and better sanitation.

The estimated map of *Culex* mosquitoes demonstrated the higher risks associated with the rice ecosystems and rural areas that were observed in some rice growing areas of My Duc, Phuc Tho and Ha Dong districts (in strong red color areas in Fig 3). For rural areas, it is likely that poor environmental sanitation facilitates the mosquitoes’ breeding habitats whereas people in urban areas are more aware of JE virus and other vector-borne diseases. For instance, one study in Pakistan showed that 65% of urban households implemented mosquito control measures, while only 39% of rural households did it [58]. However, previous studies in the Hanoi Province have had mixed findings. Jakobsen et al suggested that peri-urban households in Hanoi had better mosquito control knowledge and practices compared to households in more central districts [59]. Other studies have suggested that regular indoor spraying and removing water containers close to the house could contribute to a reduction of mosquitoes and vector-borne diseases [60,61].

Over the past two decades, remote sensing has increasingly involved in mapping mosquito-borne risks and species distribution. Michael et al highlighted that increasingly available earth observation data play a crucial role in the control of vector-borne diseases [62]. In addition, Anna et al indicated that remote sensing-based variables were likely to reduce the over-fitting problems in ecological statistical models [63]. Several global and regional mappings of mosquito-borne diseases and species distribution took advantage of this unique data nature and provided useful information [34,64,65]. Such data visualization would be very important to clinicians to target vulnerable areas and early intervention programs are timely undertaken for those areas.

Although coarse spatial resolution data has been frequently used in geospatial public health, it is less likely to take into consideration local variations or micro-habitats because of large pixel size [24–26]. For example, an earlier Asian JE and *Culex* mosquito risk map showed the spatial patterns of mosquitoes and vector-borne diseases but it did not identify the accurate locations where mosquito-borne diseases occur (e.g. at village level) [26]. This limitation was improved in our study by constructing 30 x30m pixel size raster layer and computing variations among local pixels (e.g. using a 250m buffer) as this better resolution can reflect local variations and consequently increase the accuracy of mosquito risk maps [66].

Our study was not able to identify the geographic association between JE reported cases and *Culex* mosquitoes because JE data was unavailable. However, several studies showed that *Culex* mosquito abundance was geographically associated with JE, and West Nile viruses [25,26,67]. In Vietnam, *Culex* mosquitoes are the primary vector for JE virus across the country [14,28]. From a public health point of view, risk maps can be useful to increase the public awareness in areas with high-risk of *Culex* mosquitoes. This early warning information can alert people to take precautions such as wearing long-sleeved shirts, trousers, and hats when engaged in outdoor activities or travelling to hotspots. *Culex* mosquitoes lay their eggs in standing water, such as is found in rice fields and septic tanks, but can also breed in smaller water bodies. Our risk map identified some high-risk areas with more than 130 mosquitoes/ 900m^2^ in the rice fields and rural areas where further preventive control measures need to be considered (e.g. use of insecticides and proper sanitation).

This study had several limitations. First, the mosquito sampling was conducted in six out of 30 districts for a limited time, so our samples may not be representative across the entire year and/or seasons. However, our study was more robust than other similar studies in Egypt, Italy, and the United States that employed one-period sampling of mosquitoes, in terms of large mosquito sampling points collected and/or higher spatial resolution [68–71]. In addition, we may have overestimated the mosquito population in this study because samples were collected during the wet season (May–October), not the dry season (November–April). One study suggested that *Culex* mosquitoes were more predominant (43%) during the rainy season than the dry season in Hanoi [12]. We assumed that more mosquitoes have a higher chance of being infected with JE virus. However, we did not know the proportion of mosquitoes with JE virus as laboratory confirmation was not conducted. Furthermore, livestock are commonly raised in many rural/peri-urban districts of Hanoi, but this raster layer was not available for our study. Previous research suggested that livestock production was associated with the mosquito abundance and JE virus [72,73]. Animal waste and nearby ponds can provide favorable mosquito-breeding areas if the livestock and their barns were not regularly washed and cleaned [74,75]. Lastly, the human population density used in this study was from 2015, which was assumed to be constant for 2018, but it was unlikely to be accurate because of rapid urbanization in Hanoi.

Our findings may provide better insight into understanding the spatial distribution of *Culex* mosquitoes and its associated environmental risk factors. Such information can assist local clinicians and public health policymakers to identify the potential areas of risk for JE virus. Risk maps can be an efficient way of raising public awareness about the virus and further preventive measures need to be considered in order to prevent outbreaks and onwards transmission of the JE virus.
